# Corrosion Resistance of Selective Laser Melted Ti6Al4V3Cu Alloy Produced Using Pre-Alloyed and Mixed Powder

**DOI:** 10.3390/ma15072487

**Published:** 2022-03-28

**Authors:** Gonghao Huang, Zefeng Fan, Liu Li, Yanjin Lu, Jinxin Lin

**Affiliations:** 1 College of Chemistry, Fuzhou University, Fuzhou 350108, China; huanggonghao@fjirsm.ac.cn (G.H.); fanzefeng@fjirsm.ac.cn (Z.F.); liliu0229@126.com (L.L.); 2 Key Laboratory of Optoelectronic Materials Chemistry and Physics, Fujian Institute of Research on the Structure of Matter, Chinese Academy of Sciences, Fuzhou 350002, China; 3 Fujian Science & Technology Innovation Laboratory for Optoelectronic Information of China, Fuzhou 350108, China

**Keywords:** selective laser melting, Ti6Al4V3Cu, corrosion behavior, ion release, passive film

## Abstract

Metallic elemental powder mixture and pre-alloyed metallic powder are both frequently used powder feedstock in the additive manufacturing process. However, little research has been conducted to compare the corrosion behavior of selective laser melting (SLM) alloys, fabricated by pre-alloyed metallic powder and mixed metallic powder. Hence, it is important to investigate the corrosion behavior of SLMed alloys, as well as the corresponding cast ingot, with the aim to better understand the feasibility of designing new materials. In this work, the SLM-produced Ti6Al4V3Cu alloys were manufactured using a metallic elemental powder mixture and pre-alloyed metallic powder, respectively. The corrosion behavior of the different Ti6Al4V3Cu alloys was investigated in following electrochemical tests and ion release measurements. The results showed that the Ti6Al4V3Cu alloy prepared by pre-alloyed metallic powder showed better corrosion resistance than that produced from mixed metallic powder. Moreover, the SLM-produced Ti6Al4V3Cu alloys performed significantly better in corrosion resistance than the cast Ti6Al4V3Cu. The results are expected to achieve a better understanding of the feasibility of designing new materials using mixed powders, contributing to reducing development costs and cycles.

## 1. Introduction

Titanium and its alloys are commonly utilized to manufacture dental implants because of its superior mechanical properties, corrosion resistance, and biocompatibility. However, the inflammatory lesions around implants are collectively recognized as peri-implant diseases and are widespread [1]. There were almost 500 species of bacteria in the human oral microbiome, which were easily colonized on different implants [2]. Peri-implantitis was more commonly associated with opportunistic infections, such as *Pseudomonas aeruginosa* and *Staphylococcus aureus* (*S. aureus*) in observational studies [3]. According to clinical data, peri-implantitis can start early, since the majority of implants showed symptoms of bone loss (>0.5 mm), after the second (52 wt.%) and third (66 wt.%) years of use. These figures were 70 wt.% and 81 wt.% at the issue level, respectively [4]. Although many measures have been used to prevent bacterial infection, such as disinfection and sterilization during surgery, a bacterial infection often occurs in the process of recovery after surgery. Therefore, preventing and treating peri-implantitis caused by bacterial infection is a grave problem in dental implants. Antibiotics are the main treatment for peri-implantitis after scaling/root planning or mechanical debridement [5]. However, antibiotic treatment is reported to promote the generation of resistant organisms, increase the treatment cost and pollute the environment [6]. To overcome the above shortcomings, researchers have focused on the development of antibacterial biomaterials. Alloying silver (Ag) and copper (Cu) in titanium-based alloys, for example, is a potential strategy for reducing or eliminating peri-implantitis [7]. The Cu element, one of the essential cofactors of many enzymes, is used as a potent and broad-spectrum antibacterial alloying element to design antibacterial alloys [8]. Given this, various kinds of Cu-bearing alloys, such as Ti-Cu [9,10], CoCr-Cu [11,12], and 316-Cu [13], have been developed by casting, powder metallurgy and additive manufacturing, etc.

As one of the metal additive manufacturing procedures, selective laser melting (SLM) can employ a specialized scanning strategy to melt the pre-deposited metallic powder, layer by layer, in a sequential manner [14]. In this regard, SLM has been widely used to fabricate the implant with a complex structure that patients need directly. Extensive studies have been reported, showing that the titanium-based implants produced by SLM exhibit excellent mechanical properties, compared with their traditionally manufactured counterparts [15], resulting from the rapid solidification-induced refined microstructure [16,17]. F.B et al. confirmed that, due to the high cooling rate, the presence of harder microstructural constituents in Ti6Al4V, produced by SLM, leads to a higher wear resistance than hot pressing and casting specimen [18]. Thanks to the manufacturing flexibility, SLM is a facile means to design and predict new materials using mixed powders, which can reduce development costs and cycles compared with traditional methods [19]. As a result, mixed metallic powder has been recognized as a possible alternative raw material for SLM, as well as a commonly used powder feedstock in the SLM process. For example, Wang et al. investigated the homogenization of microstructure and phases of an SLM-produced Ti-35Nb composite (in wt.%), using elemental powder [20,21]. Qin et al. used high purity elemental titanium and copper powder, after mechanically mixing, to produce the solid Ti-5Cu samples and showed that heat-treated Ti-5Cu samples had similar corrosion behavior to pure CP-Ti [22]. Fan et al. demonstrated that the TC4-5Cu/TC4 alloy produced by SLM exhibited significant antibiofilm property against S. mutans [23]. In our previous study, a series of Cu-bearing Ti6Al4V alloys were fabricated by selective laser melting (SLM) technology with mixed powders, consisting of Cu and Ti6Al4V, and the effects of Cu content on the microstructure, corrosion resistance, antibacterial properties, and cytotoxicity of SLM-produced Ti6Al4V-Cu alloys were systematically assessed [24,25]. Based on the above results, Ti6Al4V3Cu pre-alloyed powders were produced using the gas atomization method, from the corresponding cast ingot, and then used in the SLM technology [26]. Further, the corrosion resistance of the SLM-produced Ti6Al4V3Cu alloys, produced from the pre-alloyed powder, was investigated at different heat treatments.

As a rule, corrosion resistance is greatly associated with mechanical properties and biocompatibility. To date, there are few studies concerning the comparison between the SLM parts produced by the use of mixed powders and corresponding pre-alloyed powder. Qin et al. compared Ti35Nb alloys fabricated by the mixed powder and pre-alloyed powder, and the corrosion behavior of the two specimens was identical, and double-layered oxide films could be formed on both SLM-produced Ti35Nb samples, from different powder feedstock [27]. Although we investigated the corrosion behavior of the SLM-produced Ti6Al4V3Cu alloys made from pre-alloyed powders, the potentially different corrosion behavior between the Ti6Al4V3Cu alloys, made from the two types of powders as powder feedstock, was overlooked, limiting a better understanding of the feasibility of designing new materials using mixed powders. This is because the mixed metallic powder after SLM processing, resulting in a non-uniform microstructure with heterogeneous distribution of the Cu element, may probably deteriorate the mechanical properties and corrosion behavior [28].

In this study, the mixed metallic powder and pre-alloyed metallic powder were used to prepare SLM-produced Ti6Al4V3Cu alloys. To investigate the corrosion behavior of those samples thoroughly, the electrochemical experiment was determined, including electrochemical impedance spectroscopy and potentiodynamic polarization. At the same time, the traditional casting of the Ti6Al4V3Cu alloy was used as a control group. The differing passivation behavior among those alloys was illuminated. This work is expected to lay the foundation for understanding the design and development of new materials, using pre-alloyed powder and mixed powders in SLM processing.

## 2. Materials and Methods

### 2.1. Materials Preparation

**Figure 1:**
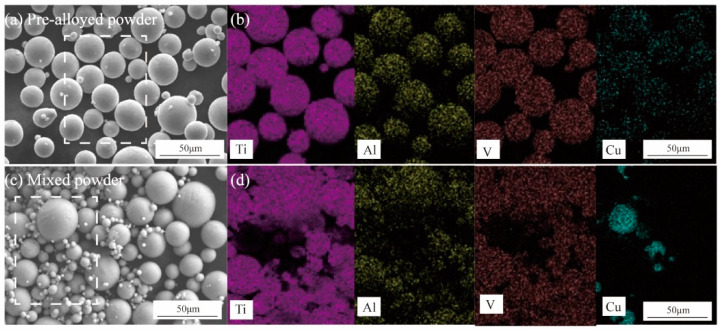
The scanning electron microscope (SEM) images of the Ti6Al4V3Cu powder: (**a**) pre-alloyed powder; (**c**) mixed powder. The pre-alloyed powder and the mixed powder of corresponding selected element distribution maps are shown in (**b**,**d**).

The pre-alloyed Ti6Al4V3Cu powder (d90: 41.39 μm) was manufactured by a TC4 and Cu ingot using gas atomization. The mixed powder (d90: 44.60 μm) was produced by mixing 97 wt.% Ti6Al4V powder and 3 wt.% Cu powders in tubular rotation. A laser particle size analyzer was used to roughly measure the powders’ distribution. Mixed elemental Ti6Al4V3Cu powder and pre-alloyed Ti6Al4V3Cu were utilized to feed an SLM machine (Mlab Cusing R, Concept Laser GmbH, Lichtenfels, Germany), that utilized a 95 W Nd: YAG laser. The images of Ti6Al4V3Cu powder using a scanning electron microscope are shown in Figure 1. During the SLM process, the optimized processing parameters—laser power of 75 W, scan line hatch spacing of 110 μm, powder bed layer thickness of 25 μm, scanning speed of 0.75 m/s, liner scanning strategy of between 90—were all carefully managed. Ingot casting was used to create Ti6Al4V3Cu ingot alloys. In a prior investigation, we discovered that Ti6Al4V alloys made using the SLM technique and heated to 750 °C perform well. Following that, the samples were heated to 750 °C in tube furnace. After 2 h of heat preservation, those samples were followed by air quenching. The SLM-produced samples referred to casting Ti6Al4V3Cu as Ti6Al4V3Cu-C, mixed Ti6Al4V3Cu powder as Ti6Al4V3Cu-M, and pre-alloyed Ti6Al4V3Cu powder as Ti6Al4V3Cu-P.

### 2.2. Materials Preparation

Before microstructural analysis, all samples were ground with SiC emery papers up to 2000 grits. After that, samples were polished to a 1 μm finish diamond paste. Then the samples were etched in Kroll reagent (6 mL HF + 10 mL HNO_3_ + 50 mL H_2_O). A scanning electron microscope (Hitachi SU8010 SEM, Tokyo, Japan) with an energy dispersive spectroscopy was used to examine the microstructure (EDS). Using a Cu K irradiation, the powders’ phase constitution and Ti6Al4V3Cu alloys were evaluated by X-ray diffraction (XRD, D/MAX-2500PC, Tokyo, Japan). Electron backscatter diffraction (EBSD) measurements were carried out, the EBSD data was post-processed using the HKL Channel 5 software.

### 2.3. Electrochemical Test

The potentiodynamic polarization and electrochemical impedance spectroscopy (EIS) were performed on an electrochemical working station (Gamry REFERENCE 600+, Warminster, PA, USA), a Gamry Instruments Company Global software. The corrosion resistance of different alloy samples was evaluated by a normative three-electrode. The electrochemical measurements were carried out at 37 ± 1 °C using a 0.9 wt.% NaCl solution as the working electrode and the samples as the reference electrode (WE), A saturated calomel electrode (SCE) as a reference electrode, and a platinum sheet counter electrode were the other electrodes (CE). The open-circuit potential (OCP) vs. time curve was used for 1 h, so that the stabilized potential value can be seen. After that, electrochemical impedance spectroscopy (EIS) was performed with a frequency range of 10^−2^ to 10^5^ Hz and an amplitude of 10 mV. The potentiodynamic polarization curves were measured at a scan speed of 1 mv/s, and range from −0.5 V to 2.0 V after the EIS test. For each group, three specimens were tested. The electrochemical test results were also examined using Gamry Instruments Echem Analyst software (Version 7.03). Tafel fitting of the experimental data yielded the corrosion potential (E_corr)_ and corrosion current density (I_corr_).

### 2.4. Static Immersion Test

The samples with dimensions of 10 × 10 × 10 mm^3^ were fabricated by SLM and casting. Static immersion experiments were carried out in polypropylene bottles for 12 days at 37 °C using a 0.9 wt.% NaCl solution with a surface area/volume ratio of 1 cm^2^/mL. All Ti6Al4V3Cu alloys were ground with waterproof paper to 2000 grits, followed by ultrasonic cleaning in acetone for 10 min, then sonication in ethanol for 10 min, and finally rinsed with ultrapure water. Immersion testing for each substance was repeated three times in duplicate. On an inductively coupled plasma atomic emission spectrometry system, metal ion concentrations discharged into NaCl solution were determined (ICP-AES, Ultima 2). The outermost surface chemical composition of the alloys was investigated using X-ray photoelectron spectroscopy (XPS, ESCALAB 250Xi, Thermo Fisher, Waltham, MA, USA). The XPS was fitted with a monochromatic Al Ka X-ray source with a 1486.6 eV energy level.

## 3. Results

### 3.1. Microstructure Analysis

**Figure 2:**
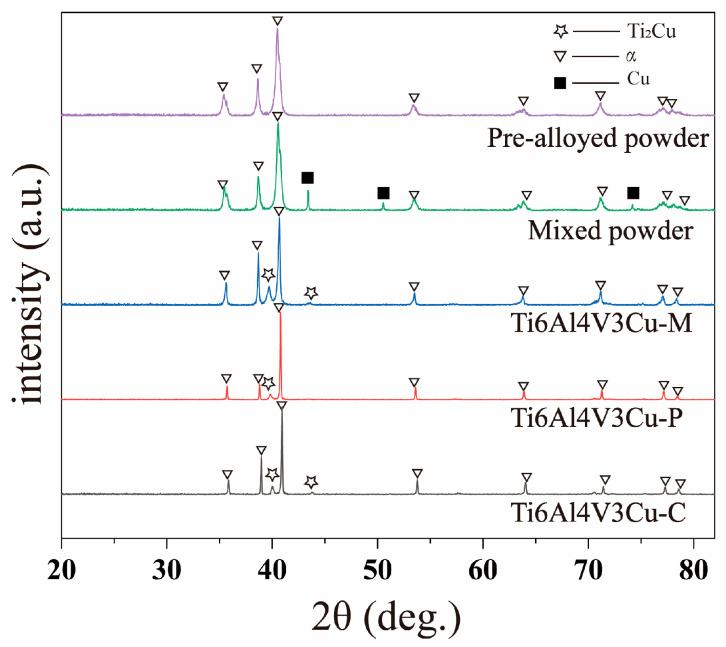
XRD patterns of the Ti6Al4V3Cu-C alloy, Ti6Al4V3Cu-P alloy, Ti6Al4V3Cu-M alloy, mixed powder, and pre-alloyed powder.

Figure 1a shows the SEM images of the pre-alloyed and mixed Ti6Al4V3Cu powders. Generally, the pre-alloyed powders are spherical in shape and range in size from 10 to 60 μm (Figure 1a). The EDS results show that the Ti, Al, V, and Cu elements are relatively evenly distributed (Figure 1b). Lots of satellite particles could be found in the mixed powders (Figure 1c), and corresponding EDS mapping suggests the copper powders are unevenly distributed in the mixed powders (Figure 1d). The XRD patterns of the powder, the corresponding SLM-produced Ti6Al4V3Cu alloys and casting alloy are illustrated in Figure 2. In all cases, the α-Ti phase was dominated in the matrix, independent of powder and bulk alloys. Moreover, the diffraction peaks of the Ti_2_Cu phase were only identified in the SLM-produced Ti6Al4V3Cu alloys and casting Ti6Al4V3Cu alloy, but not in the powders. This indicated that the Ti_2_Cu was formed during the SLM and casting processing.

**Figure 3:**
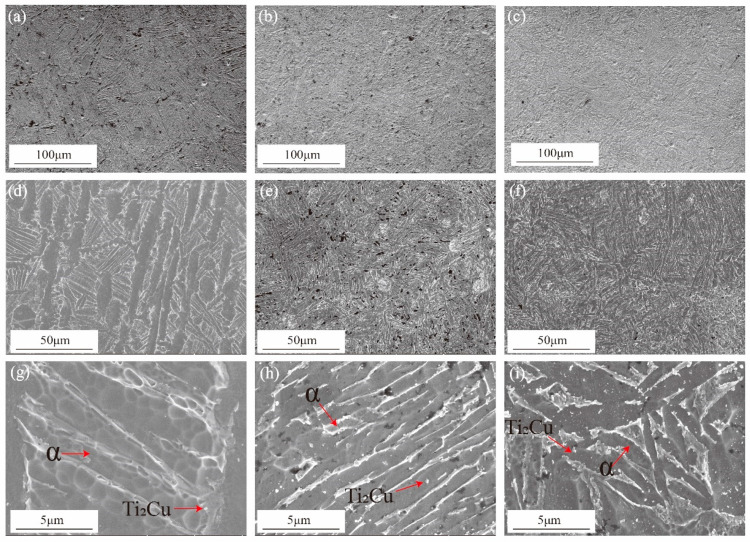
SEM images of the Ti6Al4V3Cu alloys, Ti6Al4V3Cu-C (**a**,**d**,**g**), Ti6Al4V3Cu-P (**b**,**e**,**h**), and Ti6Al4V3Cu-M (**c**,**f**,**i**).

**Figure 4:**
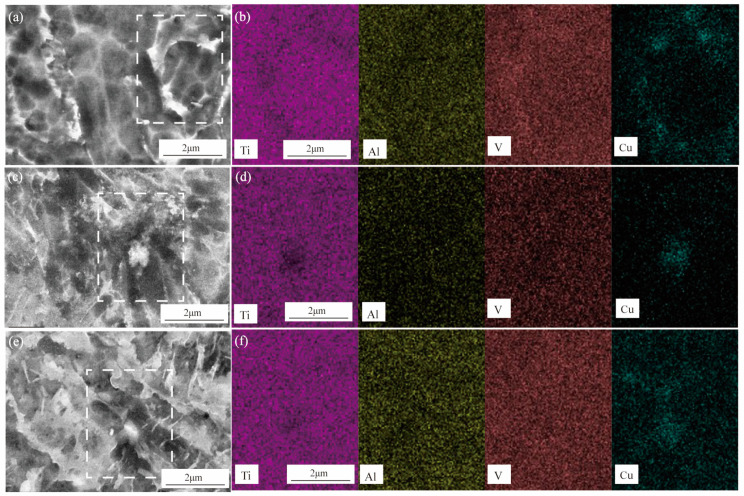
Scanning electronic microscope microstructure and mapping of alloys: Ti6Al4V3Cu-C (**a**) Ti6Al4V3Cu-P (**c**) Ti6Al4V3Cu-M (**e**) Ti, Al, V, and Cu element mapping on a Ti6Al4V3Cu-C (**b**), Ti6Al4V3Cu-P (**d**) and Ti6Al4V3Cu-M (**f**).

**Figure 5:**
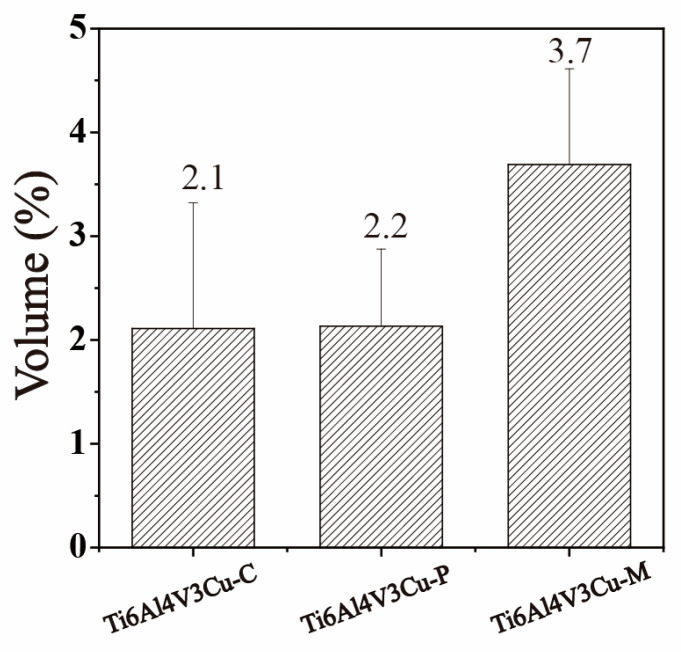
Contents of Cu element in matrix of the Ti6Al4V3Cu-C, Ti6Al4V3Cu-P, and Ti6Al4V3Cu-M alloys by EDS.

The SEM microstructure of the Ti6Al4V3Cu-P, Ti6Al4V3Cu-M, and casting Ti6Al4V3Cu alloys are shown in Figure 3. The microstructure of the casting Ti6Al4V3Cu alloy primarily consisted of a lamellar α-Ti phase structure, as shown in Figure 3a,d. In Figure 3e,f, the typical acicular martensite in the SLM-produced Ti6Al4V3Cu alloys can be observed. The resultant martensite structure was fine and accompanied by the second phase precipitation, due to the rapid melting rate in the SLM process. The microstructure, at high magnification, is shown in Figure 3g–i. In the Ti6Al4V3Cu-C alloys, the spherical and striped Ti_2_Cu precipitates nucleated at grain borders and intra-grains (Figure 3g). In the SLM-produced Ti6Al4V3Cu-P and Ti6Al4V3Cu-M (Figure 3h,i) specimens, the Ti_2_Cu phase, with a granular shape, was scattered in the grain border or within the grain. The corresponding EDS mapping of the SLM-produced and casting Ti6Al4V3Cu alloys are displayed in Figure 4, which shows that the white precipitates were rich in Cu. This further confirmed that the white precipitates in Figure 3g–i were assigned to the Ti_2_Cu phase. By calculation, the content of the Ti_2_Cu in the Ti6Al4V3Cu-M was ~7.22 wt.%, which was more than that of in the Ti6Al4V3Cu-C (~5.04 wt.%) and Ti6Al4V3Cu-P (~6.61 wt.%). Moreover, the percentage of Cu content in the matrix, determined by the EDS (Figure 5), indicated that Ti6Al4V3Cu-M exhibited the highest Cu content.

**Figure 6:**
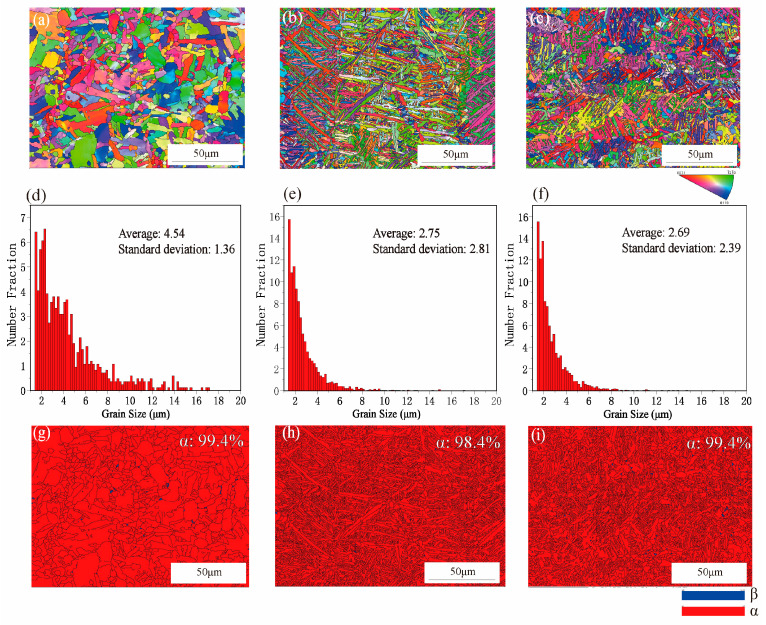
EBSD phase distribution maps of the casting Ti6Al4V3Cu alloy (Ti6Al4V3Cu-C) and selective laser-melted Ti6Al4V3Cu alloy (Ti6Al4V3Cu-P, Ti6Al4V3Cu-M) (**a**–**c**), the grain size distribution (**d**–**f**), and phase diagram (**g**–**i**).

The EBSD images in Figure 6 show the difference in microstructure between casting Ti6Al4V3Cu and SLM-produced Ti6Al4V3Cu alloys, by the phase distribution maps, the dispersion of grain sizes, and phase diagram. In the inverse pole figure in Figure 6a–c, the SLM-produced Ti6Al4V3Cu alloys showed similar misorientation angle distributions, revealing that both alloys exhibited similar textural features. The grain size in Figure 6d–f suggested that the average grain size of the Ti6Al4V3Cu-P (~2.75 μm) and Ti6Al4V3Cu-M (~2.69 μm) alloys, produced by SLM, were smaller than the casting alloys (~4.45 μm), which should be attributed to the rapid rate of melting and solidification, which caused a good grain refinement during the SLM processing [29]. Additionally, there was no significant difference in the grain size between the Ti6Al4V3Cu-P and Ti6Al4V3Cu-M alloys. The distribution of hexagonal α phase and β phase in the matrix is shown in the phase maps in Figure 6g–i, indicating that all alloys were dominated by the α phase, with a minute quantity percentage of β phase.

### 3.2. Electrochemical Analysis

**Figure 7:**
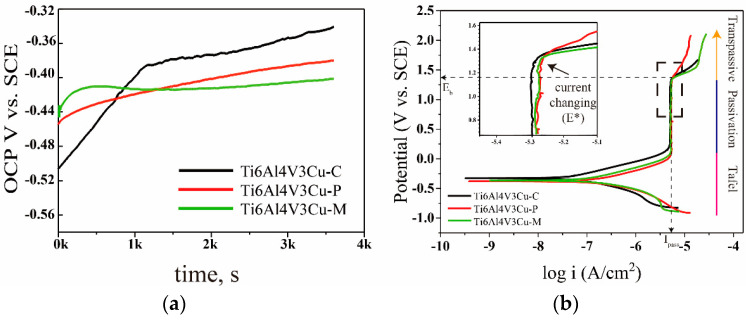
(**a**) Open-circuit potential (OCP) curves of Ti6Al4V3Cu alloys, (**b**) Typical potentiodynamic polarization curves in 0.9 wt.% NaCl solution.

Figure 7a shows the variation tendency of open-circuit potential (OCP) curves of different Ti6Al4V3Cu alloys, in 0.9 wt.% NaCl solution at 37 °C. In the case of the Ti6Al4V3Cu-C and Ti6Al4V3Cu-P alloys, the OCP curve increased quickly at initial times, then reached a steady state. Figure 7b shows the potentiodynamic polarization curves of SLM-produced and casting Ti6Al4V3Cu alloys. All potentiodynamic polarization curves showed a similar shape, consisting of the active region (pink segment), then reaching a significant passivation area (blue segment), and finally a transgressive region (yellow segment). Table 1 lists the fitted findings of the potentiodynamic polarization curves, including corrosion current density (I_corr_), corrosion potential (E_corr_), corrosion rate, and breakdown potential. As can be seen from Table 1, the Ti6Al4V3Cu-C alloy displayed the greatest corrosion rate compared with the SLM-produced ones. The corrosion current density for the Ti6Al4V3Cu-C, Ti6Al4V3Cu-P, and Ti6Al4V3Cu-M was 66.6 ± 17.6 nA, 84.2 ± 15.5 nA and 215 ± 22.51 nA, respectively. Generally speaking, the corrosion current density is a significant indicator for determining a material’s corrosion resistance. Therefore, the SLM-produced alloys showed a lower corrosion current density compared with the Ti6Al4V3Cu-C alloy, while the Ti6Al4V3Cu-P, produced by pre-alloyed powder, exhibited the lowest corrosion current density. The corrosion potential of the Ti6Al4V3Cu-C, Ti6Al4V3Cu-P, and Ti6Al4V3Cu-M was −321.7 ± 21.2 mV, −388.5 ± 10.2 mV, and −354.0 ± 42.1 mV, respectively, while the breakdown potential (E_b_) decreased, with the following order: Ti6Al4V3Cu-P > Ti6Al4V3Cu-M > Ti6Al4V3Cu-C. The breakdown potential (E_b_) is frequently used to determine the stability of passive films on metal surfaces, as an important reference for evaluating the rupture sensitivity of passivation films, and higher E_b_ values commonly signify more stable passive films [30]. Herein, the Ti6Al4V3Cu-P has the highest E_b_ value, compared to other samples. In a word, The SLM-produced Ti6Al4V3Cu alloys had superior corrosion resistance over the casting ones. Moreover, the corrosion resistance of the alloys produced from the pre-alloyed powder was greater than that produced using mixed powders.

**Figure 8:**
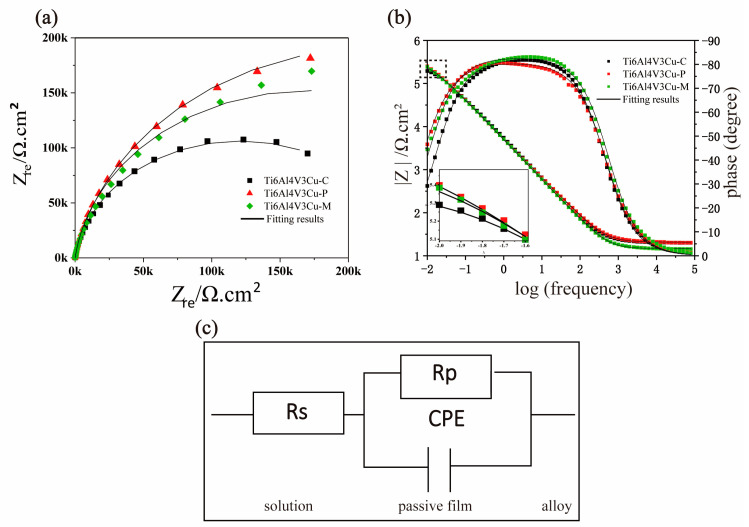
(**a**) Nyquist diagrams (**b**) Bode plots and (**c**) equivalent electrical circuit to fit the EIS test.

Figure 8a shows the EIS plots of Ti6Al4V3Cu-C, Ti6Al4V3Cu-M and Ti6Al4V3Cu-P alloys after OCP. The Nyquist plot of all alloys exhibited one single capacitive loop, independent of manufacturing methods, suggesting a similar corrosion mechanism in alloys. Moreover, the Ti6Al4V3Cu-P alloy presented the largest diameter of the capacitive arcs, whereas the least was observed in the Ti6Al4V3Cu-C alloy. The capacitive arcs of Ti6Al4V3Cu-M were in between the Ti6Al4V3Cu-C and Ti6Al4V3Cu-P. Generally speaking, a larger diameter in the corresponding semicircle represents a higher corrosion resistance. Thus, the corrosion resistance was decreased in the following order: Ti6Al4V3Cu-P < Ti6Al4V3Cu-M < Ti6Al4V3Cu-C. In the Bode plots in Figure 8b, the corresponding phase angle and impedance values have a slight reduction, in the order of Ti6Al4V3Cu-C, Ti6Al4V3Cu-P, and Ti6Al4V3Cu-M alloys, in the low-frequency range between 0.1 Hz and 0.01 Hz, indicating less resistive passive oxide films in Ti6Al4V3Cu-C alloys. SLM-produced Ti6Al4V3Cu alloys had a wider platform in the middle-frequency (100 Hz and 0.1 Hz) region compared with that produced by casting, and the slope of the line located in the middle frequency was close to −1, meaning the passivation membrane has a capacitive behavior. The maximum phase angles of Ti6Al4V3Cu-C, Ti6Al4V3Cu-P, and Ti6Al4V3Cu-M were −81.8°, −80.5°, and −83.1°, respectively, which further indicated the oxide film formed after electrochemical testing was stable and had integrity in the structure. At high frequencies, the log |Z| metric tends towards constant values, whereas phase angle values decrease to zero, indicating that the impedance was dominated by the charitable electrolyte resistance (Rel). Furthermore, the relationship between frequency and phase angle curves in Figure 8b indicated that all Ti6Al4V3Cu alloys had only one capacitive loop in the entire frequency range.

The EIS experimental results were fitted using the equivalent circuit model to quantitatively determine the electrochemical performance of the passivation membrane, as shown in Figure 8c. A constant phase element (CPE) was linked parallel to an oxide film resistance (R_p_) in the utilized EEC, and a solution resistance (R_s_) was connected in series to the preceding two components. Due to the frequency dependency of capacitance, the CPE component was used in the EEC. Surface inhomogeneity, point defect distribution, and space charge layer are possible variables. The following equation is used to compute the impedance of CPE.

(1) $$Z_{\mathrm{CPE}}=\frac{1}{Y_{0}}{\left(\mathrm{jw}\right)}^{-n}$$

In the above formula, j means the imaginary number or (−1)1/2, and ω means angular frequency. Y_0_ in this formula represented the capacitance of the electrochemical corrosion experiment system, and n, which usually lies between 0.5 and 1. The simple resistor, transferring to a pure capacitor to the CPE, is indicated by the value of n shifting from 0 to 1, and when n = 0.5, it represents a Warburg impedance. The parameters, including the R_s_, R_p_, n, and Y_0_, are summarized in Table 2. It shows that the value of n for all alloys was very close, indicating the surface of all the samples’ dissolution under hybrid control. As for the R_p_, the Ti6Al4V3Cu-P alloy presented the largest value of R_p_, whereas the smallest was observed in the Ti6Al4V3Cu-C alloy, and Ti6Al4V3Cu-M was in between Ti6Al4V3Cu-C and Ti6Al4V3Cu-P. This result reflected that the SLM-produced Ti6Al4V3Cu alloys exhibited excellent corrosion resistance, concerning the one made by casting, while the Ti6Al4V3Cu-P alloy had more stability, in terms of oxide film, than that of the Ti6Al4V3Cu-M alloy.

### 3.3. ICP Analysis

**Figure 9:**
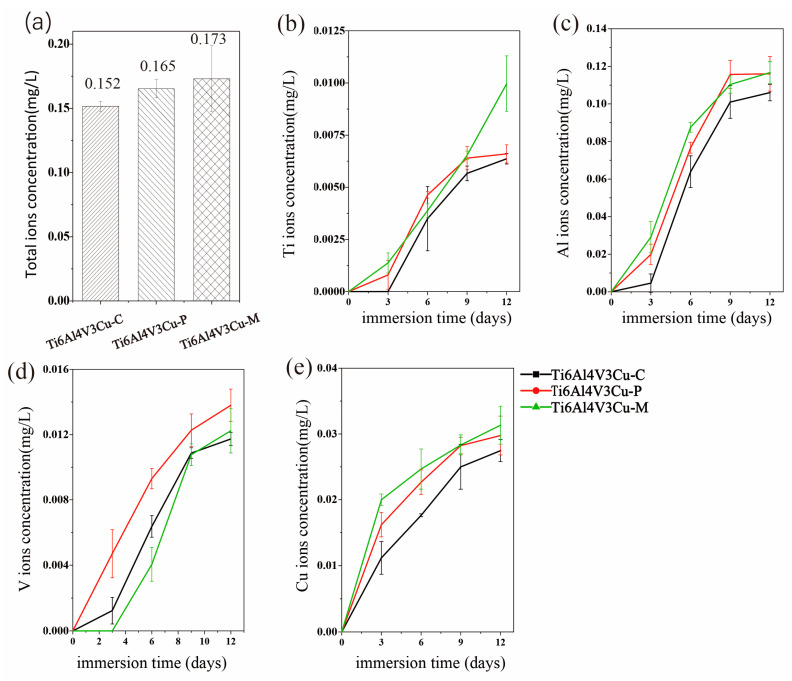
(**a**) Metal ion release by casting Ti6Al4V3Cu alloy (Ti6Al4V3Cu-C) and selective laser-melted Ti6Al4V3Cu alloys (Ti6Al4V3Cu-P, Ti6Al4V3Cu-M). The total amount of metal ions released. The release of (**b**) Ti ions, (**c**) Al ions, (**d**) V ions, and (**e**) Cu ions.

The accumulated concentration of metal ions, including the Ti, Al, V, and Cu ions, in the 0.9 wt.% NaCl, after 12 days immersion, is shown in Figure 9a. As can be seen from Figure 9a, the total ions concentration of the Ti6Al4V3Cu-C alloys (0.152 mg/L) was lowest, compared to that of Ti6Al4V3Cu-P (0.165 mg/L) and Ti6Al4V3Cu-M (0.172 mg/L). Moreover, the accumulated concentration of metal ions for Ti6Al4V3Cu-M, produced by mixed powders, was higher than that of the Ti6Al4V3Cu-P produced by pre-alloyed powders, but showed no statistical difference (*p* > 0.05). Figure 9b–e displays the Ti, Al, V, and Cu ion in the 0.9 wt.% NaCl solution, after 3 days, 6 days, 9 days, and 12 days of immersion with the samples, respectively. Generally, all ions showed a sustainable increase in profiles with time, and the concentration of Ti, Al, and Cu ions was increased, in the following order: Ti6Al4V3Cu-C < Ti6Al4V3Cu-P < Ti6Al4V3Cu-M. The concentrations of the Cu ion for Ti6Al4V3Cu-C, Ti6Al4V3Cu-P and Ti6Al4V3Cu-M were 0.027 mg/L, 0.029 mg/L, 0.031 mg/L respectively. Compared with the recommended dietary recommendations for Cu (1.3 mg), suggested by the WHO [31], the release of Cu ions from all alloys is very low [31].

### 3.4. XPS Study

**Figure 10:**
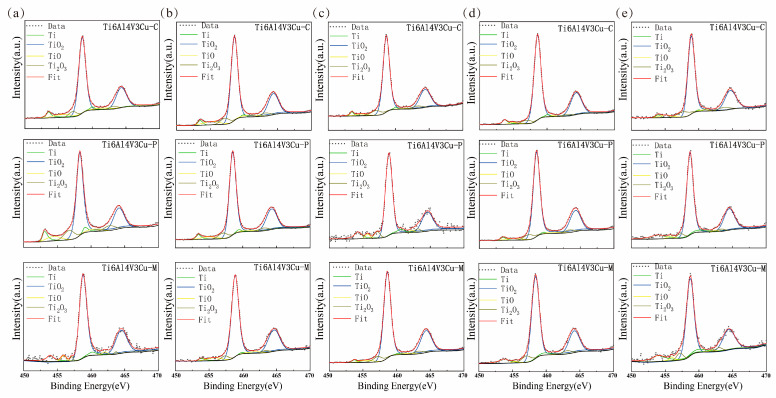
XPS survey spectra of the different Ti6Al4V3Cu alloys surface in 0, 3, 6, 9, 12 days exposure to 0.9 wt.% NaCl solution at 37 °C. high-resolution XPS spectra of Ti2p region from metal (**a**) 0-day, (**b**) 3-day, (**c**) 6-day, (**d**) 9-day, (**e**) 12-day.

**Figure 11:**
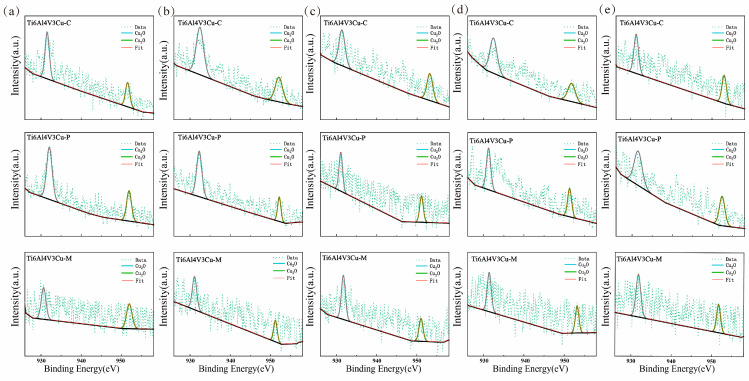
XPS survey spectra of the different Ti6Al4V3Cu alloys surface in 0, 3, 6, 9, 12 days exposure to 0.9 wt.% NaCl solution at 37 °C. high-resolution XPS spectra of Cu2p region (**a**) 0-day, (**b**) 3-day, (**c**) 6-day, (**d**) 9-day, (**e**) 12-day.

**Figure 12:**
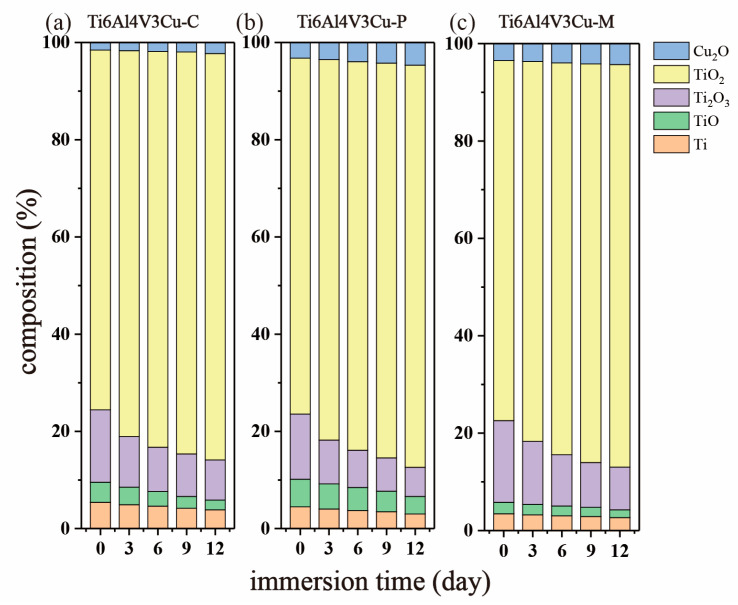
Before and after immersion in a 0.9 wt.% NaCl solution at 37 °C, the relative concentrations of Ti6Al4V3Cu-C alloys(**a**), Ti6Al4V3Cu-P (**b**), and Ti6Al4V3Cu-M (**c**) in the surface oxides.

X-ray photoelectron spectroscopy was used to determine the surface chemical composition of the passive film on Ti6Al4V3Cu-C, Ti6Al4V3Cu-P, and Ti6Al4V3Cu-M alloys, as shown in Figure 10 and Figure 11. In the case of the Ti2p spectra in Figure 10, the peaks at a binding energy of 453.7 eV and 459.4 eV were assigned to metallic Ti; the peaks located at 455.3 eV for Ti2p_3/2_ and 460.5 eV for Ti2p_1/2_ belonged to TiO; the peak at 457.2 eV for Ti2p_3/2_ and 462.9 eV for Ti2p_1/2_ was attributed to the Ti_3_O_2_, while the peaks located at 458.3 eV for Ti2p_3/2_ and 464.3 eV for Ti2p_1/2_ were assigned to TiO_2_. Figure 11 presents the Cu2p spectra for the Ti6Al4V3Cu-C, Ti6Al4V3Cu-P, and Ti6Al4V3Cu-M alloys. Generally, the peaks that appeared at the binding energies of 932.2 eV, and 952.8 eV were assigned to Cu_2_O. The composition of Ti and Cu in the passive film, calculated from the XPS spectra, is shown in Figure 12, which indicated that TiO_2_ was dominated in the passive film in all alloys, independent of manufacturing methods, and the content of TiO_2_ was found to increase with time. Moreover, after 12 days of immersion, Ti6Al4V3Cu-P contained the highest portion of TiO_2_ in the passive film. In addition, before and after immersion, the amount of the Cu_2_O in the Ti6Al4V3Cu-C alloys was the lowest, compared with the SLM-produced alloys, and there was no significant difference between the Ti6Al4V3Cu-P and Ti6Al4V3Cu-M alloys in Cu_2_O content.

## 4. Discussion

In this study, the Ti6Al4V3Cu alloys were fabricated by selective laser melting, employing pre-alloyed metallic powder and mixture elemental powder as powder feedstock, respectively, aiming to compare their corrosion behavior. The above results indicated that the SLM-produced Ti6Al4V3Cu has a better corrosion resistance compared with the casting alloy. When compared to Ti6Al4V3Cu-M alloys, Ti6Al4V3Cu-P had superior corrosion resistance. Generally, the corrosion behavior is greatly associated with the microstructure, including composition, grain size, precipitate, etc. On the one hand, the factor associated with the grain size should explain the different corrosion behavior. The β phase contains a high content of V and has been shown to improve the corrosion resistance of Ti6Al4V alloys compared to α phase [32]. The phase distribution in Figure 6g–i shows that the β phase in Ti6Al4V3Cu-P alloys, as a proportion, is the highest. Furthermore, the small grain size that resulted (Figure 6a,b) from the selective laser melting processing favored the better corrosion resistance, concerning the casting Ti6Al4V3Cu-C alloy. The nucleation rate during solidification is one of the influencing variables in grain refining, as the rapid solidification rate, up to 10^5^–10^6^ °C/s, provides a greater driving force for grain nucleation, resulting in refined grains. In addition, much finer α needles and a gradually finer structure, apparently consisting of degenerate lamella, were observed due to the higher cooling rates. This is the formation mechanism of the SLM-produced Ti6Al4V3Cu alloys’ microstructure, shown in Figure 3, while the Ti6Al4V3Cu alloys obtained by casting, were processed by a continuous casting process, where liquid metal was solidified with a specific cooling rate, thus, leading to different microstructures, such as primary α phase and α coarse lamellae (transferred from β). According to previous reports, grain refinement can increase electron activity at grain boundaries, which can help the rapid formation of stable passive films, improving corrosion resistance [33]. Dai et al. [34] proved that the size of the grain has a significant influence on the corrosion rate, and it is proposed that the corrosion rate and grain size show a positive correlation. Herein, the EBSD result shows that the grain size distribution in the Ti6Al4V3Cu-P and Ti6Al4V3Cu-M alloys was smaller than that of the Ti6Al4V3Cu-C (Figure 6d–f). This could explain why the corrosion resistance of the SLM-produced alloys showed better corrosion resistance compared with the casting alloys.

On the other hand, the distribution form of Cu should be responsible for the different corrosion behavior between the Ti6Al4V3Cu-P and Ti6Al4V3Cu-M alloys. From Figure 1a, the distribution of Cu in the pre-alloyed Ti6Al4V3Cu powders was very uniform, while it was heterogeneous in the mixed Ti6Al4V3Cu powders. The heterogeneous distribution of Cu in the mixed Ti6Al4V3Cu powders caused certain parts to be affluent in Cu, while others were deficient in Cu during melting, resulting in an inhomogeneous distribution of Ti_2_Cu [28]. Such a case could be avoided in the pre-alloyed condition. Many studies have reported that the heterogeneous presence of the intermetallic Ti_2_Cu phases generates galvanic cells, relative to the matrix in the electrolyte, as the Ti_2_Cu phases exhibit a greater standard electrode potential compared with the titanium matrix [35]. The galvanic cells would be impeded when the uniform distribution of the Ti_2_Cu phases was achieved in the matrix. W.R. Osório proved that by imposing Ti_2_Cu as a corrosion protection component, the heat treatment of the Ti–7.1 percent Cu alloy resulted in an utterly eutectoid microstructure, which reduced the galvanic corrosion impact [36]. In this study, the casting alloy shows a lath-like microstructure and a spherical Ti_2_Cu precipitate, whereas the SLM-produced alloys have alternating layers of phase and Ti_2_Cu, as well as needles that resemble a martensitic structure (Figure 3). As shown in Figure 3e,f, the content of the Cu element in the matrix of the Ti6Al4V3Cu-M alloy was greater than that of the Ti6Al4V3Cu-P alloy, indicating that the amount of Ti_2_Cu in Ti6Al4V3Cu-M was more than in Ti6Al4V3Cu-P alloys. In addition, some reports demonstrated that the Ti_2_Cu phase would influence the stability of the passive film. First, the galvanic couples, constituted by the primary α-Ti and Ti_2_Cu phase, result in a more irregular oxide film and a deterioration in corrosion resistance [37]. That is, the irregular presence of the Ti_2_Cu phase could be detrimental to the formation of a compact passive film [38]. Second, Cu_2_O, with a p-type semiconductor structure in the passive film, exhibits massive defects, including metal cation vacancy and oxygen vacancy. This defect would promote the reaction of oxygen vacancy and outward metal cation vacancy at the interface, between matrix and film, preventing further growth of the passive film. The Ti_2_Cu phase can be covered by the TiO_2_ oxide layer when the α-Ti dissolves and forms the passivation film, when the Ti_2_Cu phase is evenly distributed in the α-Ti phase. The quality of the passive film, covering the Ti_2_Cu phase, on the other hand, is determined by the Ti_2_Cu phase’s distribution. In this study, the inhomogeneous distribution of Ti_2_Cu and the Cu-rich phase in Ti6Al4V3Cu-M alloy reduced the uniformity of the passivation film, causing the corrosion resistance to be slightly worse than for the Ti6Al4V3Cu-P alloy, and the release of the Cu ion in the Ti6Al4V3Cu-M alloy is higher than the Ti6Al4V3Cu-P alloy (Figure 9). Particularly, Ti6Al4V3Cu-P, with the finer and homogeneous distribution of Ti_2_Cu phases, favored the formation of the highest content of TiO_2_, in the outermost layer, as shown in Figure 12. Therefore, the non-uniform distribution of copper is the main factor for contributing to the decreased corrosion resistance in the SLM-produced Ti6Al4V3Cu alloys, created from mixture powder, compared to the pre-alloyed metallic powder. However, it must be noted that the casting Ti6Al4V3Cu-C alloy showed the lowest ion concentration compared with the SLM-produced alloys, although the SLM-produced alloys showed the lower corrosion current density, which was reported in the previous study [26]. Therefore, the in-depth study and underlying mechanism should be explored in further study.

## 5. Conclusions

In this study, pre-alloyed metallic powder and mixed elemental powder were used as powder feedstock, to fabricate the Ti6Al4V3Cu alloy by selective laser melting, and the corresponding casting ingot was used as the control group. The potentially different corrosion behaviors were investigated by the electrochemical test and the static immersion test. The limited conclusions were obtained as follows:

1. The intermetallic compound Ti_2_Cu could be found in all microstructures of Ti6Al4V3Cu alloys, regardless of the processing method. However, the processing method could affect the distribution form of the Ti_2_Cu, and the more uniform distribution Ti_2_Cu could be found in the Ti6Al4V3Cu-P alloys.
2. Compared to the microstructures of the casting alloy, the SLM-produced Ti6Al4V3Cu alloys exhibited finer martensite and small grain size.
3. The corrosion experiment revealed that, due to the grain refinement of the SLM process, SLM-produced Ti6Al4V3Cu performs better than the casting sample, in terms of anti-corrosion capability.
4. The corrosion resistance of Ti6Al4V3Cu alloys, fabricated by pre-alloyed metallic powder, is superior to that fabricated by the mixed elemental powder because of the uniform Cu element in the matrix.

## Figures and Tables

**Table 1:** The I_corr_, E_corr_, corrosion rate and E_b_ value obtained from polarization curves.

Samples	I_corr_ (nA)	E_corr_ (mV)	Corrosion Rate (μg∙cm^−2^∙yr^−1^)	E_b_ (mV)
Ti6Al4V3Cu-C	215 ± 22.51	−321.7 ± 21.2	174.2 ± 18.1	1270 ± 4
Ti6Al4V3Cu-P	66.6 ± 17.6	−388.5 ± 10.2	53.9 ± 14.3	1359 ± 6
Ti6Al4V3Cu-M	84.2 ± 15.5	−354.0 ± 42.1	68.3 ± 12.6	1336 ± 4

**Table 2:** Equivalent circuit parameters calculated by fitting EIS experimental findings.

Samples	R_s_ (Ω·cm^2^)	R_p_ (K·Ωcm^2^)	Y_0_ (μΩ^−1^∙s^n^∙cm^−2^)	n
Ti6Al4V3Cu-C	19.91 ± 1.76	231.52 ± 25.94	29.12 ± 4.9	0.89 ± 0.03
Ti6Al4V3Cu-P	21.81 ± 2.36	371.67 ± 103.60	35.87 ± 2.86	0.91 ± 0.01
Ti6Al4V3Cu-M	17.7 ± 2.95	354.17 ± 141.78	34.09 ± 2.81	0.89 ± 0.05

## Data Availability

Not applicable.
